# Mental health disorders and readmissions following acute myocardial infarction in the United States

**DOI:** 10.1038/s41598-022-07234-z

**Published:** 2022-02-28

**Authors:** Jayakumar Sreenivasan, Risheek Kaul, Muhammad Shahzeb Khan, Aaqib Malik, Muhammad Shariq Usman, Erin D. Michos

**Affiliations:** 1 Department of Cardiology, Westchester Medical Center and New York Medical College, Valhalla, NY USA; 2 Department of Cardiology, University of Mississippi Medical Center, Jackson, MS USA; 3 Department of Internal Medicine, Dow University of Health Sciences (DUHS), Karachi, Pakistan; 4 Division of Cardiology, Johns Hopkins University School of Medicine, Baltimore, MD USA

**Keywords:** Cardiovascular diseases, Psychiatric disorders

## Abstract

Hospital readmissions following an acute myocardial infarction (MI) are associated with increased mortality and morbidity. The aim of this study was to investigate if there is a significant association between specific mental health disorders (MHD) and risk of hospital readmission after an index hospitalization for acute MI. We analyzed the U.S. National Readmission Database for adult acute MI hospitalizations from 2016 to 2017. Co-morbid diagnoses of MHD were obtained using appropriate ICD-10-CM diagnostic codes. The primary outcome of interest was 30-day all-cause unplanned readmission. Cox-regression analysis was used to identify the association of various MHD and risk of 30-day readmission adjusted for demographics, medical and cardiac comorbidities, and coronary revascularization. We identified a total of 1,045,752 hospitalizations for acute MI; patients had mean age of 67 ± 13 years with 37.6% female. The prevalence of any MHD was 15.0 ± 0.9%. After adjusting for potential confounders, comorbid diagnosis of major depression [HR 1.11 (95% CI 1.07–1.15)], bipolar disorders [1.32 (1.19–1.45)], anxiety disorders [1.09 (1.05–1.13)] and schizophrenia/other psychotic disorders [1.56 (1.43–1.69)] were independently associated with higher risk of 30-day readmission compared to those with no comorbid MHD. We conclude that MHD are significantly associated with a higher independent risk of 30-day all-cause hospital readmissions among acute MI hospitalizations.

## Introduction

Mental health has a bidirectional impact on cardiovascular health^1^. Negative psychological factors like mental health disorders (MHD) and stress can lead to a higher risk for cardiovascular diseases whereas positive affect and happiness are associated with better cardiovascular outcomes^2^. Recent United States (U.S.) nationwide data showed an increasing trend in the prevalence of MHD among patients hospitalized for acute myocardial infarction (MI)^3^. Hospital readmission following acute MI is associated with increased mortality and morbidity^4^; however, the association of MHD and readmissions following acute MI hospitalization is not well studied. In a study limited to Mental Health Research Network (MHRN)-affiliated health systems evaluating patients admitted for acute MI, pneumonia and heart failure during 2009–2011, the rate of readmission was 5% greater for individuals with a past-year psychiatric comorbidity than for those without^5^. Another 8-year follow-up study with a relatively small sample size based on the Hospital Discharge Administrative Database revealed that the impact of psychiatric disorders on readmissions for acute MI are comparable to diabetes, obesity, cerebral vascular disease, and hypertension^6^. However, there is a lack of a contemporary, all-payer national study in the U.S. on the impact of MHD and readmission following acute MI. Such a study would provide insights into the unconventional risk factors for readmission following acute MI. The aim of this study was to assess if there is a significant association between specific MHD and the risk of hospital readmission after an index hospitalization for acute MI. The main objective was to investigate a public national database in the U.S. to study the patients with a co-morbid diagnosis of major depression, bipolar disorder, anxiety disorders and schizophrenia/other psychotic disorders who were admitted with acute MI, compared to patients with MI and no MHD. The association of these MHD and the risk of hospital readmission was to be assessed over a period of 30 days following index hospitalization. We hypothesized that co-morbid diagnoses of these MHD would be associated with a significantly increased independent risk of 30-day readmission following acute MI.

## Methods

### Data source

We queried the National Readmission Database (NRD) maintained by Healthcare Cost and Utilization Project (HCUP) for the year 2016–2017 for our study. The NRD is an administrative longitudinal database representing encounter-level information on all-payer inpatient stay in the United States. It contains stratified weighted data from 20% of all non-federal acute care hospitals in the nation enabling us to analyze national estimates of inpatient hospitalizations and subsequent readmissions. NRD tracks each admission for subsequent readmissions during the calendar year by using a unique patient linkage number specific to each patient care encounter. We analyzed the data from most recently available NRD 2016-2017 to align with the contemporary clinical practice and hospitalization patterns in the management of acute MI. Our entire dataset included patient diagnosis and procedures encoded with the use of comprehensive International Classification of Diseases Tenth revision Clinical Modification/Procedure Coding System (ICD 10 CM/PCS). The ICD 10 CM/PCS is the latest revision of the globally accepted classification system for diseases and procedures that is maintained by the World Health Organization. Institutional Review Board (IRB) approval or informed patient consent for study participation were not required as NRD constitutes deidentified patient information and it is publicly available for analysis. The study was conducted in accordance with the Declaration of Helsinki and the HCUP guidelines. HCUP certification required for use of any NRD database resource for scientific studies was completed by the primary author (JS) of this study.

### Inclusion/Exclusion criteria, variables and outcome of interest

**Figure 1:**
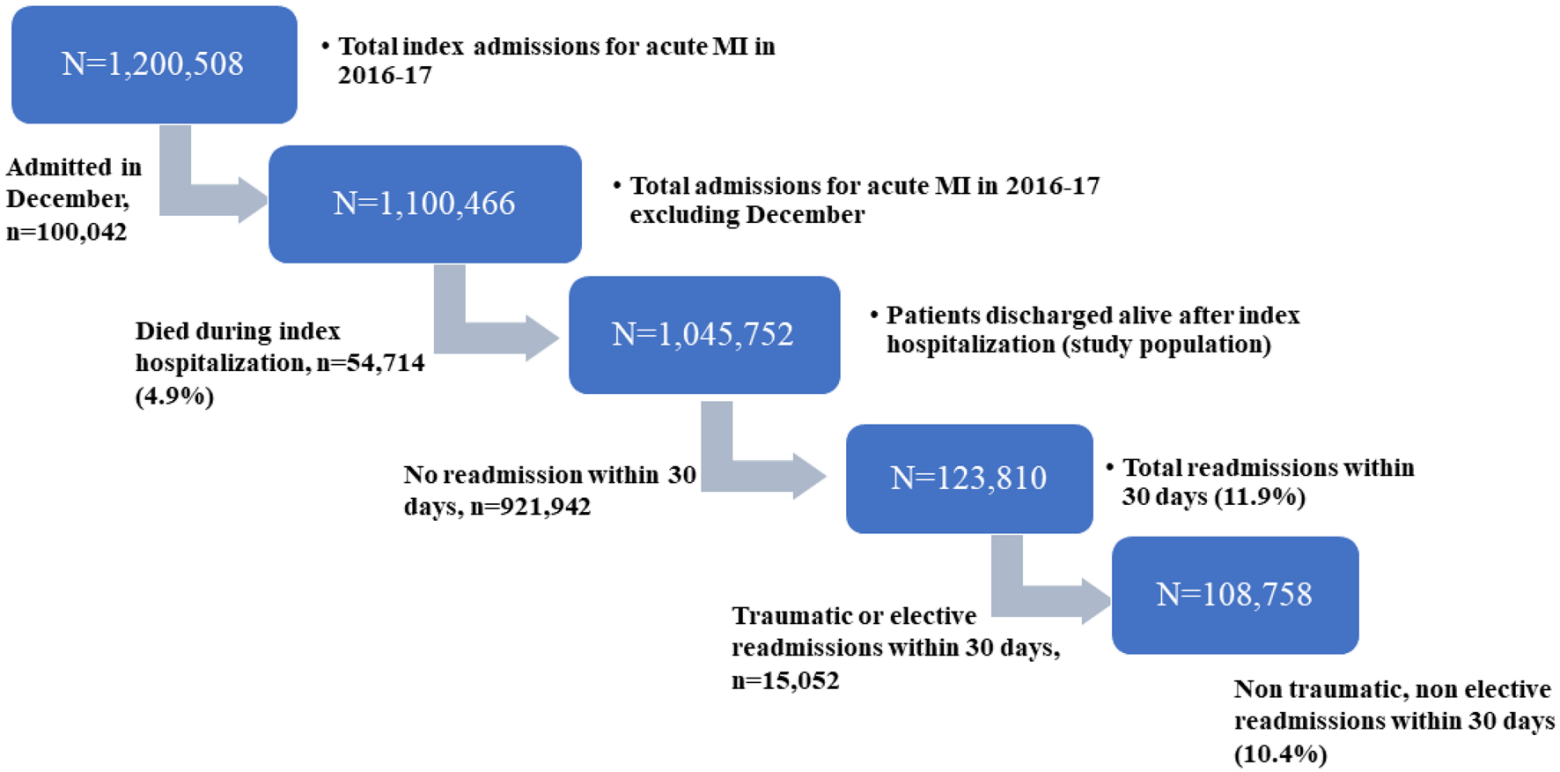
Study flow diagram.

We identified all admissions in adults (age > 18) with a primary diagnosis of acute MI (ICD 10 CM I2101, I2102, I2109, I2111, I2119, I2121, I2129, I213x I214x) during the year 2016 to 2017 in the United States. Patients admitted during the month of December were excluded since the NRD database does not track readmissions across the calendar year (Fig. 1). Patients who were readmitted following accidents, external injuries or for preplanned elective admissions within the 30 days were excluded from readmission cohort. Patients with major depression (ICD10-CM-F32x and F33x), bipolar disorder (ICD10-CM-F31x), anxiety disorders (F41x) and schizophrenia/other psychotic disorders (ICD10-CM-F2x) were identified using appropriate ICD-10 diagnostic codes. Various other medical and cardiac comorbidities of total study population were obtained using standard ICD 10 CM codes. Demographics, vital statistics and hospital characteristics were obtained from the NRD directly. The primary outcome of the study is the all-cause readmission rate within 30 days following an index hospitalization for acute MI.

### Statistical analysis

We used STATA, version 16 (StataCorp, TX) for all the statistical analyses. The NRD has a complex sampling design that includes stratification, clustering, and weighting. We have utilized appropriate discharge weights (DISCWT) using *svyset* command in STATA to account for stratification, clustering, and weighting in NRD to obtain accurate national estimates. Continuous variables were compared using Student t test and categorical variables were compared using Fischer exact test. We did survival analysis, with the primary outcome (all cause rehospitalization within 30 days) as the ‘failure event’ and time from index hospitalization discharge to first readmission as ‘time to failure event’ for evaluating the independent association of various MHD and the risk of 30-day readmission. Patients who were discharged alive and those who were not readmitted within 30 days were censored at 30 days. Univariate Cox regression analysis was performed to identify significant predictors of all cause 30-day readmissions. The variables assessed were age, sex, hospital characteristics (bed-size, location), income, insurance, medical co-morbidities including diabetes mellitus, hypertension, heart failure, prior percutaneous or surgical coronary revascularization, atrial fibrillation, chronic obstructive pulmonary diseases, congestive heart failure, atrial fibrillation, chronic liver or kidney diseases, peripheral arterial diseases and index hospital coronary revascularization. A *p* < 0.1 were used as a cut off for univariate screening of candidate predictors for inclusion in multivariate regression analysis. All significant predictors were included in the multivariate Cox Regression model for identifying independent association of MHD and the risk of 30-day readmissions. The visual inspection of the survival curves for various MHD and 30-day readmission revealed parallel curves satisfying the proportional hazard assumption. Further assessment based on scaled Schoenfeld residuals supported proportional hazards assumption by a non-significant relationship between residuals and time (*p* value = 0.46) with a zero-slope plot (Supplemental Figure 1). A sensitivity analysis was performed by evaluating sub-groups of interests based on age, sex, Medicare vs non-Medicare insurance status and index hospital coronary revascularization. The differences between groups were tested using a subgroup-MHD interaction term in the regression model and reported as *Pinteraction*. A small percent of data regarding the insurance status (3.4%) and income status (1.7%) of the patients was missing from the database. These 2 variables with missing data were tested after multivariate imputation by chained equation based on all other known variables. Results with and without imputation were not meaningfully different. A two-sided *p* value < 0.05 was considered statistically significant.

## Results

We identified a total of 1,045,752 hospitalizations for acute MI with a mean age of 66.6 ± 12.9 years with 37.6% being females (Fig. 1). The prevalence of any MHD was 15.0%. The prevalence of the MHD subtypes was 5.2% for major depression, 0.8% for bipolar disorder, 8.8% for anxiety disorder, and 0.7% for schizophrenia/other psychotic disorder. Note that the MHD subtypes were not mutually exclusive. The baseline characteristics of the study population including demographics, hospital characteristics and medical comorbidities are provided in Table 1. The patients with MHD were significantly less likely to undergo coronary revascularization (major depression 52.8%, bipolar disorder 56.7%, anxiety disorder 56.7%, and schizophrenia/other psychotic disorders 41.7%) during index hospitalization compared to those without any co-morbid MHD (61.6%) (*p* < 0.001).

**Table 1:** Baseline characteristics of study population stratified by mental health disorders.

Patient characteristics	No mental health disorders (n = 888,889)	Major depression (n = 54,379)	Bipolar disorder (n = 8,366)	Anxiety disorders (n = 92,026)	Schizophrenia and other psychotic disorders (n = 7,320)	*p* value
Mean age (years) ± SD	66.9 ± 12.3	67.3 ± 15.1	58.5 ± 16.1	64.1 ± 12.1	62.2 ± 14.2	< .001
Female (%)	34.9	51.2	45.0	55.0	38.2	< .001
Rural residential location (%)	21.2	21.6	19.1	22.2	20.6	< .001
**Type of insurance**						
Medicare (%)	59.6	66.4	56.3	58.4	64.4	< .001
Medicaid (%)	8.2	9.6	22.6	11.2	25.0	
Private (%)	27.7	20.0	15.9	25.3	6.9	
Uninsured/self-paid (%)	4.4	2.6	5.2	3.1	2.7	
Teaching hospital (%)	67.3	68.7	69.5	65.9	69.9	< .001
Urban hospital location (%)	92.9	92.7	93.8	92.3	92.8	< .001
**Income quartile for the residential ZIP code**						
0-25th percentile	28.1	28.6	34.3	30.5	39.2	< .001
26th–50th percentile	27.0	27.5	27.2	27.6	26.7	
51st–75th percentile	24.0	24.3	22.1	23.4	20.4	
76th–100th percentile	20.8	19.6	16.4	18.5	13.7	
Anemia (%)	2.8	3.3	2.8	2.9	3.6	< .001
Obesity (%)	16.5	18.3	20.1	18.6	14.3	< .001
Diabetes mellitus (%)	44.0	50.9	49.5	41.8	47.8	< .001
Hyperlipidemia (%)	56.8	62.3	57.2	62.8	46.2	< .001
Hypertension (%)	77.7	80.7	75.8	77.9	76.8	< .001
Liver disease (%)	2.4	2.5	3.5	2.3	3.8	< .001
Chronic kidney disease (%)	23.2	24.1	17.7	16.0	20.5	< .001
Hemodialysis dependence (%)	1.9	1.7	1.3	0.9	1.9	< .001
Pulmonary vascular disease (%)	4.9	4.8	3.4	3.9	4.8	< .001
COPD (%)	19.6	26.9	31.4	27.2	32.2	< .001
Peripheral arterial disease (%)	8.9	9.7	6.6	8.4	5.6	< .001
Dementia (%)	4.8	8.9	4.8	5.2	12.8	< .001
Prior MI (%)	12.5	12.8	13.2	12.4	11.5	< .001
Prior CABG (%)	8.0	7.6	5.5	5.9	4.2	< .001
Prior PCI (%)	11.9	11.1	11.0	10.5	6.5	< .001
Prior stroke (%)	5.4	6.6	5.9	5.2	6.5	< .001
Congestive heart failure (%)	39.7	40.5	35.6	34.3	43.7	< .001
PCI (%)	52.8	45.7	49.8	48.4	34.9	< .001
CABG (%)	9.6	7.6	7.4	8.9	6.6	< .001
Coronary revascularization (%)	61.6	52.8	56.7	56.7	41.7	< .001
Duration of hospitalization (days) ± SD	4.6 ± 2.2	4.4 ± 2.1	4.7 ± 2.6	4.1 ± 1.9	5.1 ± 2.6	< .001

*CABG* coronary artery bypass grafting, *COPD* chronic obstructive pulmonary disease, *MI* myocardial infarction, *PCI* percutaneous coronary intervention, *SD* standard deviation.

**Figure 2:**
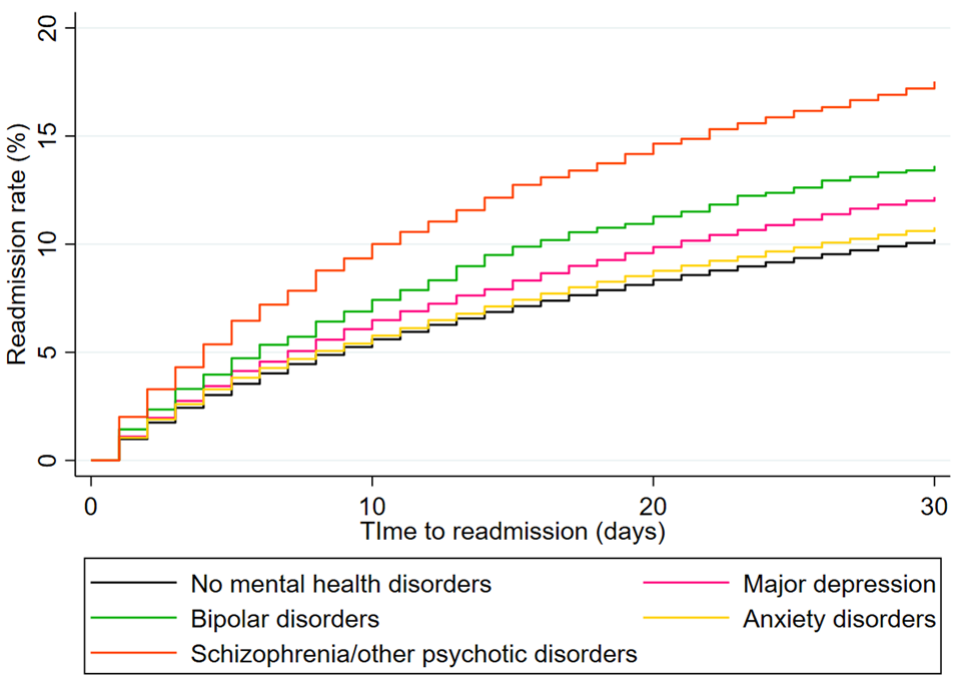
Mental health disorders and 30-day readmission rate following acute myocardial infarction. The 30-day all-cause readmissions following an index hospitalization for acute myocardial infarction categorized by various co-morbid mental health disorders compared to those without any co-morbid mental health disorders.

The 30-day all-cause readmission rate for the whole study cohort was 10.4 ± 0.3% and for patients with no comorbid MHD was 10.3 ± 0.3%. Recurrent non-ST elevation MI was the single most common reason (10.5 ± 0.8% of all readmissions) for rehospitalization. After adjusting for potential confounders, comorbid diagnosis of major depression [rate 12.2% ± 0.5%, hazard ratio (HR) 1.11, 95% confidence interval (CI) 1.07–1.15, *p* < 0.001], bipolar disorders (rate 13.6 ± 0.5%, HR 1.32, 95% CI 1.19–1.45, *p* < 0.001), anxiety disorders (rate 10.9 ± 0.4%, HR 1.09, 95% CI 1.05–1.13, *p* < 0.001) and schizophrenia/other psychotic disorders (rate 17.5 ± 0.6%, HR 1.56, 95% CI 1.43–1.69, *p* < 0.001) were independently associated with higher risk of 30-day readmission compared to those with no comorbid MHD (Fig. 2, Table 2). There were significant differences between sub-groups on sensitivity analyses and interaction testing (Table 3). Among hospitalizations for acute MI, younger patients with age < 60 years, men, non-Medicare beneficiaries and those who received index hospital coronary revascularization demonstrated a significant association between MHD and high risk of readmission compared to the other cohorts. The results of the regression analysis did not change significantly with or without multiple imputation for missing data (Table 3).

**Table 2:** Predictors of readmission after acute MI.

Variables	Univariate screening			Multivariate regression analysis		
	Hazard ratio	95% CI	*p* value	Adjusted hazard ratio	95% CI	*p* value
Age	1.02	1.01–1.03	< .001	1.01	1.002–1.003	< .001
Female sex	1.29	1.26–1.32	< .001	1.11	1.08–1.13	< .001
**Insurance**						
Medicare	Ref	Ref	Ref	Ref	Ref	Ref
Medicaid	0.90	0.88–0.93	< .001	1.09	1.05–1.13	< .001
Private	0.42	0.41–0.44	< .001	0.64	0.61–0.66	< .001
Uninsured/self-pay	0.52	0.49–0.55	< .001	0.79	0.74–0.84	< .001
**Hospital bed-size**						
Small	Ref	Ref	Ref	Ref	Ref	Ref
Medium	0.99	0.94–1.03	0.7	1.02	0.97–1.07	0.3
Large	0.95	0.91–0.99	0.02	1.03	0.98–1.07	0.2
Hospital teaching status	0.95	0.81–0.86	< .001	0.97	0.94–1.00	0.07
Urban hospital location	0.84	0.81–0.86	< .001	0.86	0.84–0.89	< .001
**Income quartile for the residential ZIP code**						
0-25th percentile	Ref	Ref	Ref	Ref	Ref	Ref
26th–50th percentile	0.91	0.88–0.94	< .001	0.95	0.92–0.97	< .001
51st–75th percentile	0.87	0.84–0.89	< .001	0.91	0.88–0.94	< .001
76th–100th percentile	0.81	0.78–0.85	< .001	0.84	0.81–0.88	< .001
Hypertension	1.29	1.24–1.31	< .001	0.98	0.96–1.01	0.3
Diabetes mellitus	1.42	1.40–1.45	< .001	1.17	1.15–1.19	< .001
Anemia	1.54	1.47–1.61	< .001	1.33	1.20–1.44	< .001
Obesity	0.81	0.79–0.83	< .001	0.86	0.76–0.96	< .001
Prior CABG	1.27	1.23–1.31	< .001	1.07	1.04–1.10	< .001
Prior PCI	0.97	0.92–1.00	0.05	0.99	0.96–1.02	0.6
Prior stroke	1.26	1.22–1.30	< .001	1.11	1.08–1.15	< .001
Congestive heart failure	2.06	2.03–2.10	< .001	1.58	1.54–1.62	< .001
Atrial fibrillation	1.45	1.42–1.49	< .001	1.19	1.17–1.23	< .001
Dementia	1.38	1.33–1.43	< .001	0.99	0.95–1.02	0.5
Pulmonary vascular disorders	1.66	1.61–1.72	< .001	1.11	1.07–1.14	< .001
Chronic liver disorders	1.45	1.38–1.52	< .001	1.36	1.30–1.43	< .001
Chronic kidney disease	2.09	2.05–2.14	< .001	1.46	1.43–1.49	< .001
Hemodialysis dependence	2.44	2.34–2.55	< .001	1.47	1.40–1.54	< .001
Peripheral arterial disease	1.37	1.33–1.41	< .001	1.13	1.09–1.16	< .001
Chronic obstructive pulmonary disease	1.49	1.46–1.52	< .001	1.24	1.21–1.27	< .001
Index hospital coronary revascularization	0.58	0.57–0.59	< .001	0.81	0.79–0.83	< .001
**Mental health disorders**						
Major depression	1.20	1.15–1.25	< .001	1.11	1.07–1.15	< .001
Bipolar disorder	1.35	1.22–1.50	< .001	1.32	1.19–1.45	< .001
Anxiety disorders	1.06	1.03–1.09	< .001	1.09	1.05–1.13	< .001
Schizophrenia/other psychotic disorders	1.79	1.64–1.95	< .001	1.56	1.43–1.69	< .001

*CABG* coronary artery bypass grafting, *PCI* percutaneous coronary intervention.

**Table 3:** Sensitivity analyses by specific sub-groups for the association of hospital readmission after acute myocardial infarction.

Variables	Multivariate regression analysis			
	Adjusted hazard ratio	95% CI	*p* value	*Pinteraction*
**Age ≥ 60 years**				< .001
Major depression	1.06	1.02–1.07	.006	
Bipolar disorder	1.21	1.07–1.38	.003	
Anxiety disorders	1.05	1.01–1.08	.01	
Schizophrenia/other psychotic disorders	1.44	1.29–1.61	< .001	
**Age < 60 years**				
Major depression	1.21	1.12–1.31	< .001	
Bipolar disorder	1.42	1.25–1.61	< .001	
Anxiety disorders	1.22	1.16–1.29	< .001	
Schizophrenia/other psychotic disorders	1.70	1.49–1.94	< .001	
**Female sex**				< .001
Major depression	1.04	0.98–1.09	0.1	
Bipolar disorder	1.24	1.08–1.41	.002	
Anxiety disorders	1.02	0.98–1.06	.2	
Schizophrenia/other psychotic disorders	1.36	1.19–1.56	< .001	
**Male sex**				
Major depression	1.16	1.09–1.22	< .001	
Bipolar disorder	1.39	1.22–1.58	< .001	
Anxiety disorders	1.19	1.13–1.24	< .001	
Schizophrenia/other psychotic disorders	1.68	1.51–1.86	< .001	
**Medicare patients**				< .001
Major depression	1.06	1.02–1.10	.006	
Bipolar disorder	1.15	1.03–1.30	.02	
Anxiety disorders	1.05	1.01–1.09	.004	
Schizophrenia/other psychotic disorders	1.45	1.30–1.60	< .001	
**Non-Medicare patients**				
Major depression	1.18	1.09–1.26	< .001	
Bipolar disorder	1.59	1.38–1.82	< .001	
Anxiety disorders	1.18	1.12–1.25	< .001	
Schizophrenia/other psychotic disorders	1.64	1.43–1.89	< .001	
**With index hospital coronary revascularization**				< .001
Major depression	1.18	1.11–1.24	< .001	
Bipolar disorder	1.30	1.14–1.49	< .001	
Anxiety disorders	1.15	1.10–1.20	< .001	
Schizophrenia/other psychotic disorders	1.69	1.48–1.94	< .001	
**Without index hospital coronary revascularization**				
Major depression	1.05	1.01–1.10	.03	
Bipolar disorder	1.32	1.16–1.49	< .001	
Anxiety disorders	1.04	1.01–1.09	.04	
Schizophrenia/other psychotic disorders	1.43	1.28–1.59	< .001	
**With imputation of missing data**				0.1
Major depression	1.11	1.08–1.16	< .001	
Bipolar disorder	1.33	1.20–1.48	< .001	
Anxiety disorders	1.09	1.05–1.14	< .001	
Schizophrenia/other psychotic disorders	1.60	1.48–1.76	< .001	
**Without imputation of missing data**				
Major depression	1.11	1.07–1.15	< .001	
Bipolar disorder	1.32	1.19–1.45	< .001	
Anxiety disorders	1.09	1.05–1.13	< .001	
Schizophrenia/other psychotic disorders	1.56	1.43–1.69	< .001	

## Discussion

In this contemporary nationwide observational study on the impact of MHD on hospital readmissions following acute MI, we found that patients with major depression, bipolar disorders, anxiety disorders and schizophrenia/other psychotic disorders are less likely to receive coronary revascularization during index hospitalization for acute MI and they are at a significantly increased independent risk for readmission within 30 days.

Our study finding of the average rate of 30-day all-cause readmission at 10.4% is consistent with prior national estimates of hospital readmission rate following acute MI^7^. The added risk of readmission among patients with MHD is concerning especially as there is a significant increase in the prevalence of MHD among acute MI hospitalizations in the United States^3^. Depression and anxiety disorders are associated with a higher risk of recurrent adverse cardiac events following acute MI^8–10^. Previous history of depression or anxiety is associated with significant emotional distress post-MI which can potentially lead to adverse clinical outcomes^11^. The population-based studies suggest that one-third of the mortality among patients with bipolar disorders are due to cardiovascular diseases^12^ and these patients carry a higher independent risk for recurrent cardiac events and mortality at short-term follow-up after acute MI^13^. Even though studies on schizophrenia and post MI outcomes are limited, a recent study has found patients with schizophrenia to be less likely to receive guideline-directed treatment for MI and they had double the risk of adverse outcomes including readmission and mortality following MI compared to those who do not have schizophrenia^14^. These evidences suggest that the patients with MHD are at higher baseline risk for recurrent cardiovascular events which can be aggravated further by lack of good socio-economic support, poor medication adherence and difficulty in coordinating outpatient follow-up care for institutionalized patients with severe MHD. In addition, as notable in our analysis, the lower rate of index hospital coronary revascularization for acute MI among patients with MHD is another major reason leading to readmission for recurrent events and complications.

Our study reiterates the need for comprehensive care for patients with MHD hospitalized with acute MI. These patients are less likely to receive guideline-directed coronary interventions as substantiated in our study and higher risk of nonadherence to prescribed medication after discharge^13,15^. Despite calls for the increased awareness of the detrimental impact of MHD on cardiovascular health, coronary revascularization rates in patients with MHD presenting with acute MI significantly lags behind compared to that of patients with no MHD. Even though the role of medical and/or behavioral interventions to mitigate the disparities in outcomes for patients with MHD following acute MI are limited^16,17^, multidisciplinary integrated care and support is essential to reduce adverse outcomes in this high-risk population. In the current time where the hospital reimbursements, quality metrics and financial penalties are linked to unplanned readmissions^18^, it is important to focus on non-conventional risk factors like MHD as independent risk factors for readmission to further reduce the national readmission rates following acute MI.

Our study has certain limitations. Diagnosis of MHD was based on administrative billing codes and our database does not include detailed data on the severity and timing of diagnosis of these co-morbid MHD among acute MI hospitalizations. Also, the database does not include data on medical therapy and hence the readmission rates could not be adjusted for medical therapy in regression analysis. However, we have accounted for coronary revascularization during the index hospitalization in all analyses.

## Conclusion

Major depression, bipolar disorders, anxiety disorders and schizophrenia/other psychotic disorders are significantly associated with a higher independent risk of 30-day all-cause hospital readmissions following acute MI hospitalizations in the United States. Our findings further highlight the link between mental health and cardiovascular health.

## Supplementary Information

Supplementary Information.
